# Supplementary material for: Grain neighbour effects on twin transmission in hexagonal close-packed materials
Source: Nat Commun. 2016 Dec 19;7:13826. doi: 10.1038/ncomms13826 (PMC5187439; doi:10.1038/ncomms13826)
Supplement: Supplementary Information — Supplementary Note and Supplementary References. [file ncomms13826-s1.pdf]

Supplementary Note 1

Mathematical formulation: In this work, we employ an elasto-visco-plastic based Fast Fourier Transform (EVP-FFT) model[1] , which has been extended recently to account for the twin shear transformation within a discrete lamella.[2]. In this model, the elasto-visco-plastic constitutive behavior of a material under an infinitesimal strain with shear transformation, here twinning, is written as

$$\boldsymbol{\sigma}(\mathbf{x})=\mathbf{C}(\mathbf{x}):\boldsymbol{\varepsilon}^{el}(\mathbf{x})=\mathbf{C}(\mathbf{x}):\left(\boldsymbol{\varepsilon}(\mathbf{x})-\boldsymbol{\varepsilon}^{pl}(\mathbf{x})-\boldsymbol{\varepsilon}^{tr}(\mathbf{x})\right) \tag{1}$$

where $\boldsymbol{\sigma}(\mathbf{x})$ is the Cauchy stress, $\mathbf{C}(\mathbf{x})$ is the elastic stiffness tensor, and $\boldsymbol{\varepsilon}^{el}(\mathbf{x})$ is the elastic strain at a material point **x**. The elastic strain can be written as the difference between the total strain $\boldsymbol{\varepsilon}(\mathbf{x})$, and the plastic strain $\boldsymbol{\varepsilon}^{pl}(\mathbf{x})$ due to crystallographic slip and the transformation strain $\boldsymbol{\varepsilon}^{tr}(\mathbf{x})$. The plastic strain due to slip is defined as,

$$\dot{\varepsilon}^{pl}(\mathbf{x})=\sum_{s=1}^{N}\mathbf{m}^{s}(\mathbf{x})\dot{\gamma}^{s}(\mathbf{x}) \tag{2}$$

$$\dot{\gamma}^{s}(\mathbf{x})=\dot{\gamma}_{o}\left(\frac{\left|\mathbf{m}^{s}(\mathbf{x}):\boldsymbol{\sigma}(\mathbf{x})\right|}{\boldsymbol{\tau}_{c}^{s}}\right)^{n}\times\operatorname{sgn}\left(\mathbf{m}^{s}(\mathbf{x}):\sigma(\mathbf{x})\right) \tag{3}$$

where $\dot{\gamma}^{s}$ and τ_{c}^{s} are the shear rate and the critical resolved shear stress associated with each of slip system 's', respectively. Within the twin lamella, the transformation strain is given by

$$\Delta\boldsymbol{\varepsilon}^{tr}(\mathbf{x})=\mathbf{m}^{tw}(\mathbf{x})\,\Delta\gamma^{tw}(\mathbf{x}) \tag{4}$$

and outside of it, it is zero. In the above equations, $\boldsymbol{m}^{s}$ and $\boldsymbol{m}^{tw}$ represent respectively the Schmid tensor for a slip and twinning system. The integer *n* is the stress exponent. For material points **x** within the twin region, the twin transformation is achieved via N^{twincr} increments of shear strain, according to

$$\Delta\gamma^{tw}(\mathbf{x}) = \frac{s^{tw}}{N^{twincr}} \tag{5}$$

until the characteristic twinning shear s^{tw} is reached. The number of increments used to complete the twin transformation N^{twincr} is set sufficiently high to ensure convergence.

Simulation procedure: This tri-crystal model setup is subjected to compression along the y-direction (see Fig. 4), which is perpendicular to the c-axis of the parent grain. Along the x and z directions, we enforce zero average stress. This 3D stress state corresponds to a Schmid factor of ~0.5 for the (01-12)[0-111] tensile twin variant modeled. The initial compression is continued until the twin plane resolved shear stress (TRSS) is equal to or slightly greater than the CRSS. Afterwards, the twin lamella is introduced, while the unit cell is under the applied strain state, by performing the following two steps: first, the set of voxels pre-selected for the twin domain are reoriented and, next, the local twinning shear is enforced within these voxels in several increments (refer Eq. (5)). The 'strain hold' boundary condition will lead to some applied stress relaxation following the twin transformation, and is appropriate for simulating grains that are embedded in the bulk of the aggregate. In the twin shear transformation simulations, the applied strains are small and therefore work hardening and lattice re-orientations due to crystallographic slip are not crucial. However, these effects could easily be taken into account into this model should larger strain simulations be desired.

Limitations of the model: The foregoing model has the following inherent limitations. First, it does not explicitly account for the atomic scale phenomena, which would be important. The formulation does not account for size. Also, the tri-crystal is idealized such that the grain boundaries and twin boundaries are planar and flat. Last, the twin studied is stationary and subsequent propagation of this twin and the attendant re-distribution of the local stresses are not considered.

Supplementary references

1. R.A. Lebensohn, A.K. Kanjarla, P. Eisenlohr, "An elasto-visco-plastic formulation based on Fast Fourier transforms for the prediction of micromechanical fields in polycrystalline materials", *Int J of Plasticity* **32-33**, 59-69 (2012)

2. M. Arul Kumar, A.K. Kanjarla, S.R. Niezgoda, R.A. Lebensohn, C.N. Tomé, "Numerical study of the stress state of a deformation twin in magnesium", *Acta Mater* 84, 349-358 (2015)
